# Early post-transplant complications following ABO-incompatible kidney transplantation

**DOI:** 10.15171/jnp.2016.04

**Published:** 2015-12-30

**Authors:** Hamza Naciri Bennani, Zhyiar Abdulrahman, Asma Allal, Federico Sallusto, Antoine Delarche, Xavier Game, Laure Esposito, Nicolas Doumerc, Bénédicte Debiol, Nassim Kamar, Lionel Rostaing

**Affiliations:** ^1^Department of Nephrology and Organ Transplantation, CHU Rangueil, Toulouse, France; ^2^Department of Urology, Andrology, and Transplantation, CHU Rangueil, Toulouse, France; ^3^Etablissement Français du Sang de Midi-Pyrénées, CHU Purpan, Toulouse, France; ^4^INSERM U563, IFR–BMT, CHU Purpan, Toulouse, France; ^5^Université Paul Sabatier, Toulouse, France

**Keywords:** Kidney transplantation, Bleeding, Surgical complications, BK virus, Blood transfusion, Fibrinogen

## Abstract

*Background:* Living-kidney transplantation is increasing because of the scarcity of kidneys from deceased donors and the increasing numbers of patients on waiting lists for a kidney transplant. Living-kidney transplantation is now associated with increased long-term patient- and allograft-survival rates.

*Objectives:* The purpose of this retrospective study was to identify, in a cohort of 44 ABO-incompatible (ABOi) live-kidney transplant patients, the main complications that occurred within 6 months post-transplantation, and to compare these findings with those from 44 matched ABO-compatible (ABOc) live-kidney transplant patients who were also from our center.

*Patients and Methods:* This single-center retrospective study assessed post-transplantation complications in 44 ABO-i versus 44 matched ABO-c patients. All patients were comparable at baseline except that ABO-i patients had greater immunological risks.

*Results:* During the 6-month post-transplant period, more ABO-i patients presented with postoperative bleeds, thus requiring significantly more blood transfusions. Bleeds were associated with significantly lower values of fibrinogen, platelets, prothrombin time, and hemoglobin levels. Surgical complications, patient- and graft-survival rates, and kidney-function statuses were similar between both groups at 6 months post-transplantation.

*Conclusions:* We conclude that impairment of hemostatic factors at pre-transplant explained the increased risk of a post-transplant bleed in ABO-i patients.

**Implication for health policy/practice/research/medical education:**

ABO-incompatible (ABOi) live-kidney transplantation allows increasing the total number of kidney transplantation. It is associated in the long-term with similar results to those obtained with ABO-compatible (ABOc) live-kidney transplantation provided pre-transplant desensitization that includes immunosuppressive drugs and apheresis in order to remove isoagglutinins that might result in acute humoral rejection. However, pre-transplant apheresis might result in coagulation disorders which may lead to peri-transplant bleedings.

## 1. Background

Living-kidney transplantation is increasing because of the scarcity of kidneys from deceased donors and the increasing numbers of patients on waiting lists for a kidney transplant. Living-kidney transplantation is now associated with increased long-term patient- and allograft-survival rates (1). However, in the setting of living-kidney transplantation ABO-incompatibility is often an issue but can be circumvented by placing potential ABO-incompatible (ABOi) patients in a swap program (2,3) or by conducting pre-transplant desensitization (based on apheresis plus immunosuppression) to minimize the risk of acute humoral rejection (4-6).

Many registries/studies show that long-term patient- and kidney-allograft-survival rates for ABOi living-kidney patients are as good as those for ABO-compatible (ABOc) living-kidney transplant patients (5,7). However, in the early post-transplant period ABOi patients face more complications. It is reported that ABOi patients have more bleeding episodes either during transplantation or in the immediate post-transplant period (8-12). This has been largely ascribed to apheresis session(s) that the patient may have immediately before transplantation (9,10,13). Significantly more lymphoceles (which require specific therapies) as well as significantly more wound dehiscences have been reported in ABOi patients (14).

In addition to these surgical complications, registries and studies show that ABOi patients have a significantly increased incidence of BK-virus (BKV) infection, as well as BKV-associated nephropathy (14-17). Thus, compared to human leukocyte antigen (HLA)-incompatible kidney recipients, who also receive an immunosuppressive regimen at both pre-transplant and post-transplant (very similar to the treatment of ABOi patients), twice as many ABOi patients have a BKV infection compared to ABOc patients (15). The reasons underlying this complication are as yet unknown. Conversely, there is no increased risk of cytomegalovirus (CMV) infection in ABOi patients.

In 2011, we started an ABOi kidney-transplant program at our center: so far, we have treated 44 cases. The originality of this study is that the same medical team monitored patients at both pre- and post-transplantation, and that we placed special emphases on pre-transplant coagulation disorders that could be potentially caused by apheresis and might contribute to bleeds during or immediately after kidney transplantation.

## 2. Objectives

The purpose of this retrospective study was to identify, in a cohort of 44 ABOi patients, the main complications that occurred within 6 months post-transplantation, and to compare these findings with those from 44 matched ABOc live kidney transplant patients who were also from our center.

## 3. Patients and Methods

This single-center, case-controlled, retrospective study was performed in a French hospital department of nephrology and organ transplantation. We included all ABOi living-kidney transplant recipients (n = 44, 27 males, mean age of 44.7 ± 13.5 years) who attended this department between April 2011 and June 2015. The 44 patients were matched regarding gender, age, and time of transplantation, with 44 ABOc patients who were also recipients of a living kidney (i.e., 27 males, mean age of 45.2 ±13.1 years).

We collected all pertinent data that could have contributed to early peri- or postoperative (i.e., <6 months) complications. Information collected included surgical complications, bleeding episodes, rejection episodes, infections, and any metabolic disorders. The following data were also collected from both groups: donors’ and recipients’ demographic characteristics, HLA and ABO types, the donor’s renal function, HLA matching, the recipient’s original kidney disease, HLA sensitization in the recipient, warm and cold ischemia times, the need for a blood transfusion during and/or after surgery, the need for surgery to manage a complication, the presence of fluid around the transplanted kidney, any infectious complications (bacterial, viral, fungal, or parasitic), the occurrence of an acute or chronic rejection and their subtypes according to Banff 2013 criteria, and renal and metabolic post-transplant parameters.

The following parameters were assessed at regular intervals and collected at post-transplant: hemoglobin (Hb) levels, leucocyte and platelet counts, prothrombin time, fibrinogen, activated cephalin time, serum creatinine, CMV and BKV DNAemias, BKV viruria, and tacrolimus trough levels. In addition, for the ABOi group, we assessed and collected data on isoagglutinin titers at pre- and post-transplant, and on tacrolimus levels at pre-transplant.

### 3.1. Immunosuppression

Immunosuppression differed across the two groups. In ABOc patients, the induction therapy was based on basiliximab 20 mg IV (day [D]0 and D4) unless the patient was highly sensitized, i.e., panel-reactive alloantibodies >25%. In that event, basiliximab was replaced by thymoglobulin (1.25 mg/kg on D0, D2, and D4). In addition, patients received (*i*) tacrolimus at 0.2 mg/kg/d to achieve trough levels of between 7 and 10 ng/ml between D0 and D30, and between 5 and 7 ng/mL thereafter; (*ii*) mycophenolic acid (MPA) at 720 mg/b.i.d. or mycophenolate mofetil (MMF) at 1g/b.i.d. between D0 and D15, with doses halved thereafter; and *(iii)* steroids (methylprednisolone IV 10 mg/kg, 2 mg/kg, and 1 mg/kg on D0, D1, and D2, respectively, which were then rapidly tapered to 10 mg/d on D30 and to 5 mg/d on D90).

For ABOi patients, immunosuppression was begun at pre-transplant; i.e., rituximab 375 mg/m² on D30 pre-transplant, and conventional immunosuppression was started at 12 days pre-transplant, i.e., tacrolimus (0.15 mg/kg/d aiming at trough levels of 7–10 ng/ml), MPA (360 mg/b.i.d.) or MMF (500 mg/b.i.d.), plus prednisone (0.5 mg/kg/d).

In addition to these treatments, according to the isoagglutinin titers at 30 days pre-transplant, we did or did not add apheresis sessions. If the specific isoagglutinin titer was <1/8 no apheresis was performed; if the isoagglutinin titer was between 1/8 and 1/16, the patients received plasmapheresis sessions to decrease titers to <1/8. For titers that were between 1/32 and <1/128, patients were given specific immunoadsorption (Glycorex^Ò^ column, Lund, Sweden) with or without double-filtration plasmapheresis (DFPP). In cases where the isoagglutinin titer was ≥1/128, we always started desensitization with 4 DFPP sessions, which were followed (if necessary) by specific immunoadsorption in order to achieve an isoagglutinin titer on the day of transplantation of ≤1/8. In a few cases where ABOi patients had pre-transplant donor-specific alloantibody (ies) with a mean fluorescence intensity of ≥3000, we replaced DFPP and/or specific immunoadsorption with semi-specific immunoadsorption (Adasorb^Ò^ or Globaffin^Ò^ reusable columns, Fresenius, Bad Homburg, Germany).

Induction therapy based on Thymoglobulin^Ò^ (1.25 mg/kg on D0, D2, and D4) was given to cases where there were associated DSAs, ABO-incompatibility, and to the first 14 ABOi patients. Thereafter, ABOi patients received basiliximab (20 mg on D0 and D4).

Post-transplant immunosuppression relied on tacrolimus (0.2 mg/kg/d aiming at trough levels of between 8 and 12 ng/ml until D15, which was then reduced to 5–8 ng/ml), MPA (720 mg/ b.i.d.) or MMF (1g/b.i.d.), which was given until D15 and then doses were halved; plus steroids (methylprednisolone 10 mg/kg on D0, 2 mg/kg on D1, 1 mg/kg on D2, and then prednisone at 0.5 mg/kg until D10, which was then progressively tapered to attain 5 mg/d by D90).

### 3.2. Prophylaxis

If the donor was sero-CMV positive and the recipient was sero-CMV negative, then valganciclovir (900 mg/d adapted to estimated-glomerular filtration rate [eGFR]) was given for 6 months. In there was a seropositive CMV recipient, valganciclovir prophylaxis (900 mg/d, adapted to eGFR) was given for 3 months to ABOc patients and for 6 months to ABOi patients. With regards to *Pneumocystis jirovecii* prophylaxis, we gave sulfamethoxazole/trimethoprim (400 mg/80 mg) every other day for 6 months to ABOc patients and for 12 months to ABOi patients.

### 3.3. Ethical issues

The study protocol was in accordance with the Declaration of Helsinki and was approved by the Ethics Committee of Toulouse university hospital (France).

### 3.4. Statistical analysis

The results are expressed as their means (SD) or medians (ranges). Comparisons between continuous variables were made using the χ² test; comparisons between discontinuous variables were made using Student’s *t* test. Statistical significance was set at *P *= 0.05.

## 4. Results

The two groups of 44 patients were comparable with regards to the donors’ characteristics (age, inulin clearance) and the recipients’ characteristics (age, gender ratio, etiology of end-stage renal disease, warm ischemia time). However, ABOi patients had significantly more HLA mismatches compared to ABOc patients (5.16 ± 2.13 versus 3.93 ± 2.64; *P *= 0.018). In addition, significantly more ABOi patients had pre-transplant DSAs and higher panel-reactive alloantibodies; cold ischemia time was also significantly longer (293 ± 83 vs. 245 ± 61 minutes, respectively; *P *= 0.002) (Table 1).

**Table 1:** Baseline demographic data from two living-kidney transplant groups

	**ABOi patients(n=44)**	**ABOc patients(n=44)**	**P value**
Donor age (years)	48.8 ± 13	51.1 ± 10.3	NS
Donor measured GFR (mL/min)	99.1 ± 17	94.4 ± 13.9	NS
Recipient gender ratio (male/female)	27/17	27/17	NS
Recipient age (years)	45 ± 13.5	45.2 ± 13	NS
Etiology of ESRD			NS
CGN (%)	25	43	
Inherited nephropathy (%)	37.3	25	
Diabetes (%)	9.2	4.5	
Others (%)	28.5	27.5	
ABO incompatibility (n):			
A→O	33	NA	
A→B	1	NA	
B→O	4	NA	
B→A	1	NA	
AB→(O, A or B)	5	NA	
Isoagglutinin titers (medians)			
30 days pre-transplant			
Anti-A	1/32/ (1/2-1/80)	NA	
Anti-B	1/20 (1-1/128)	NA	
After transplantation			
Anti-A	1/2 (1-1/32)		
Anti-B	1/2 (1-1/40)		
HLA mismatches (A/B/DR/DQ)	5.16 ± 2.13	3.93 ± 2.64	0.018
Anti-HLA antibodies (n; %)	19 (43.2)	7 (16)	0.005
Donor-specific alloantibodies (%)	13 (29.5)	4 (9.1)	0.028
Cold ischemia time (min)	293 ± 83	245 ± 61	0.002
Warm ischemia time (min)	67 ± 23	61 ± 25	0.23
BUN (mmol/L)	18.1 ± 9.6	23.3±10	0.004

Abbreviations: NA, not applicable; ESRD, end-stage renal disease; CGN, chronic glomerulonephritis, HLA, human leukocyte antigen; GFR, glomerular-filtration rate; BUN, blood urea nitrogen; ABOi, ABO-incompatible; ABOc, ABO-compatible.

In most cases of ABOi, the donor was group A (33/44) donating to a group-O recipient. Mean isoagglutinin titers at 30 days pre-transplant were higher for anti-A (1/32: range: 1/2–1/80) than for and anti-B (1/20: range: 1–1/28) patients. On the day of transplantation, isoagglutinin titers were similar at 1/2 for anti-A and anti-B.

In the ABOi group, the number of patients with anti-HLA antibodies was significantly higher than in the ABOc group (19 versus 7; *P *= 0.005). In addition, significantly more ABOi patients had donor-specific alloantibodies (DSAs), i.e., 13 versus 4 patients in the ABOc group (*P *= 0.028).

Survival of patients was excellent in both groups (100%), and only one allograft was lost (i.e., one in the ABOi group due to renal-vein thrombosis). The percentages of patients presenting with delayed graft function, defined as serum creatinine at D5 of >250 µmol/l, was 29.5% in the ABOi group and 31.8% in the ABOc group (N.S). At least one session of postoperative hemodialysis was needed by 11.4% and 6.8% (N.S) of those in the ABOi versus the ABOc group, respectively.

Pre-transplant serum-creatinine and urea levels were significantly lower in the ABOi group (although the differences were not clinically significant). At various post-transplant periods (day 7, months 1, 3, and 6) there were no statistically significant differences in serum-creatinine levels between the two groups, although values were always numerically higher in the ABOi group (Table 2).

**Table 2:** Post-transplant patient and allograft outcomes

	**ABOi patients (n=44)**	**ABOc patients (n=44)**	**P value**
Patient survival at M6	44 (100%)	44 (100%)	NS
Graft survival at M6	43 (97.7%)	44 (100%)	NS
Delayed graft function (serum creatinine >250 µmol/L at D5 (%))	29.5	31.8	NS
Hemodialysis at posttransplant (n≥1; %)	11.4	6.8	NS
Serum creatinine (µmol/L)			
D0	563 ± 172	655 ± 243	0.045
D7	198 ± 135	203 ± 164	NS
M1	149 ± 128	131 ± 158	NS
M3	146 ± 80	118 ± 31	NS
M6	161 ± 133	121 ± 37	NS
Tacrolimus/CsA (n; %)	44 (100)	38 (86.4)	0.026
Tacrolimus trough levels (ng/mL)			
D0	8.5 ± 4.2	NA	
D7	8.2 ± 3	8.4 ± 2.9	NS
M1	9.7 ± 3	10.1 ± 6	NS
M3	8.9 ± 3.4	7.8 ± 2.6	NS
M6	7.2 ± 2.4	6.7 ± 3	NS
Induction therapy (n; %)			
ATG	27 (61.4)	6 (13.6)	<0.05
Basiliximab	17 (38.6)	34 (77.3)	<0.05
Acute humoral rejection	5 (11.3)	2 (4.5)	NS
Acute cellular rejection	4 (9)	5 (11.3)	NS
Chronic humoral rejection	1 (2.2)	1 (2.2)	NS

Abbreviations: M, month; D, day; ATG, antithymocyte globulins; NA, not applicable; CsA, cyclosporine A; ABOi, ABO-incompatible; ABOc, ABO-compatible.

Immunosuppression varied slightly between the two groups: significantly more patients received antithymocyte globulin as an induction therapy in the ABOi group compared to the ABOc group (61.4% versus 13.6%, respectively; *P* < 0.05). Consequently, significantly less ABOi patients received basiliximab as an induction therapy (38.6% versus 77.3%; *P* < 0.05). Significantly more ABOi patients had immunosuppression that relied on tacrolimus (100%) compared to only 86.4% in the ABOc group (*P* = 0.026). At post-transplant, tacrolimus trough levels did not significantly differ across the two groups.

At 6-month post-transplant antibody-mediated rejection was similar across the two groups (11.3% for ABOi versus 4.5% for ABOc patients), and similarly for acute cellular-rejection episodes (9% versus 11.3%; *P* = N.S) and chronic antibody-mediated rejection (2.2% in both groups, respectively).

### 4.1. Post-transplant surgical complications

Twelve ABOi patients (27.3%) presented with serious intra-operative bleeds compared to only 8 (18.2%; *P* = 0.0004) in the ABOc group. In addition, two ABOi patients (vs. no ABOc patients) presented with intraoperative leakage of a renal-artery anastomosis. Thus, 14 ABOi patients (vs. three ABOc patients; *P* = 0.006) required peri-transplant red blood-cell transfusions. Overall perioperative bleeding complications occurred in 20 ABOi patients compared to only 8 ABOc patients (*P *= 0.006). A post-transplant large-wound hematoma was observed in 16 ABOi patients versus 6 ABOc patients (*P *= 0.013) (Table 3).

**Table 3:** Bleeding complications and requirements for a blood transfusion

	**ABOi patients (n=44)**	**ABOc patients (n=44)**	**P value**
Overall bleeding complications (n, %)	20 (40.4)	8 (18.2)	0.006
Renal-artery anastomosis leakage (n; %)	2 (4.5)	0	NS
Intraoperative bleeding (n; %)	12 (27.3)	2 (4.5)	0.0004
Large wound hematomas (n; %)	16 (36.3)	6 (13.6)	0.013
Gross hematuria after postoperative D5 (n; %)	3 (6.8)	2 (4.5)	NS
No. of patients requiring red-blood cell transfusions between D0 and D10 (n; %)	35 (79.5)	12 (27.3)	0.00002
Intraoperative BT (n; %)	14 (31.8)	3 (6.8)	0.006
D0 BT excluding intraoperative (n; %)	12 (27.3)	3 (6.8)	0.023
D1 BT (n; %)	10 (22.7)	4 (9.1)	NS
Number of BTs/patient	3.5 ± 3.4	0.8 ± 1.9	0.00002
D0 (pre-op) fibrinogen (g/L)	1.88 ± 0.62	3.65 ± 1.62	0.0006
D0 platelets (pre-op/mm^3^)	146520 ± 35880	195610 ± 50940	0.00001
D0 (pre-op) prothrombin time (%)	83.8 ± 12.1	89.8 ± 9.5	0.012
D0 (pre-op)activated cephalin time(s)	27.1 ± 4.1	29.5 ± 4	0.005
D0 Hb (pre-op) (g/dL)	10.1 ± 1.2	10.9 ± 1.5	0.006

Abbreviations: D, day; BT, blood transfusion, i.e., red blood-cell packs; Hb, hemoglobin; ABOi, ABO-incompatible; ABOc, ABO-compatible.

There was no significant difference between the two groups (three versus vs. two) regarding persisting gross hematuria beyond day 5. Overall, bleeding complications meant that 12 ABOi patients needed a mean of 3.5 (± 3.4) red blood-cell transfusions on D0 (excluding the intraoperative period) compared to three ABOc (*P *= 0.023) patients who needed a mean of 0.8 ± 1.90 transfusions (*P *= 0.0002).

The increased number of bleeding complications in ABOi patients may have been associated with the preoperative values for fibrinogen, platelet counts, Hb levels, prothrombin time, and activated cephalin time. All these were significantly lower in ABOi patients compared to ABOc patients (Table 3).

The following surgical complications were observed (Table 4). There were no incidences of renal-artery thrombosis but one of renal-vein thrombosis within 24 hours post-transplantation, which led to a nephrectomy in an ABOi patient. One ABOi patient had an early peritoneal breach that required re-intervention. Urinary leakage was observed in two ABOc patients, who were treated by endoscopic stenting. Significant lymphoceles, i.e., leading to urinary-tract compression, were observed in 8 ABOi patients and 7 ABOc patients (N.S). Five ABOi patients presented with wound dehiscence, which required surgery in three cases; two ABOc patients had a wound dehiscence with one needing surgery. Mild renal-artery stenosis developed in 4 ABOi patients and in 6 ABOc patients.

**Table 4:** Post-transplant surgical complications

	**ABOi patients (n=44)**	**ABOc patients (n=44)**	**P value**
Renal artery thrombosis (n)	0	0	NS
Renal venous thrombosis (n)	1	0	NS
Renal artery stenosis (n; %)	4 (9.1)	6 (13.6)	NS
Urinary leakage (n; %)	0	2 (4.5)	NS
Lymphoceles (n; %)	8 (18.2)	7 (16)	NS
Wound dehiscence (n; %), requiring surgery (n)	5 (11.3), 3	2 (4.5), 1	NS
Peritoneal breach (n; %)	1 (2.3)	0	NS

Abbreviations: ABOi, ABO-incompatible; ABOc, ABO-compatible.

### 4.2. Infectious complications

Overall, infectious complications were significantly more prevalent in ABOi patients (72.7% of cases) than in ABOc patients (47.7%; *P *= 0.016), as well as cases of pneumopathy (4 vs. 0; *P *= 0.04). Conversely, incidences of sepsis, overall bacterial infections, fungal infections, and acute pyelonephritis were similar across the two groups (Table 5). BKV infection occurred more frequently in ABOi patients (18.2%) than in ABOc patients (4.5%; *P *= 0.043), however, this did not translate into significantly more BKV-associated nephropathies (three versus zero, respectively).

**Table 5:** Post-transplant infectious complications.

	**ABOi patients (n=44)**	**ABOc patients (n=44)**	**P value**
Overall infectious complications (n; %)	32 (72.7)	21 (47.7)	0.016
At least one episode of BKV viremia (n, %)	8 (18.2)	2 (4.5)	0.043
BKV nephropathy (n; %)	3 (6.8)	0	NS
CMV infection (n; %)	2 (4.5)	3 (6.8)	NS
Overall bacterial infections (n; %)	24 (54.5)	17 (38.6)	NS
Acute pyelonephritis (n; %)	6 (13.6)	7 (16)	NS
Bacterial pneumopathy (n; %)	4 (9.1)	0	NS
Sepsis (n; %)	5 (11.3)	4 (9.1)	NS
Fungal infections (n; %)	3 (6.8)	0	NS

Abbreviations: BKV, BK virus; CMV, cytomegalovirus; ABOi, ABO-incompatible; ABOc, ABO-compatible..

### 4.3. Hematological and metabolic parameters

Platelet counts were significantly lower on postoperative days 1, 2, and 7, and month 1 in the ABOi group compared to the ABOc group (Table 3). Prothrombin time was significantly lower in ABOi patients on day 1 (80.5 ± 11.2% versus 85.5 ± 8.1%; *P *= 0.018), but this difference was no longer statistically different by postoperative days 2 and 7.

Hb levels were significantly lower in ABOi patients on postoperative day 1 (10 ± 1.28 versus 10.9 ± 1.38; *P *= 0.0009) and on day 2 (9.78 ± 1.22 versus 10.47 ± 1.4 g/dl; *P *= 0.017), but this ceased to be statistically different by postoperative day 7.

Leucocyte counts were similar across the two groups at all time-points except for D1 (14912 ± 5548 versus 12112 ± 3890; *P *= 0.007) and month 3 (5767 ± 3655 vs. 6715 ± 2500; *P *= 0.0003) for ABOi and ABOc patients, respectively. Within the first 6 months post-transplant, 6 ABOi patients and 5 ABOc patients presented with one episode of leukopenia, i.e., white blood-cell counts were <1500/µl and required treatment with granocyte-stimulating factors.

*De novo* post-transplant mellitus, as defined by the need for hypoglycemic medication, occurred in 4 patients (two from each group). Post-transplant high blood pressure that required at least one medication was observed in 28 ABOi patients and 22 ABOc patients (N.S). Deep-vein thrombosis occurred in 9 ABOi patients and 7 ABOc patients (N.S). Diarrhea that lasted for more than 3 weeks occurred in 4 ABOi patients and 2 ABOc patients (N.S).

## 5. Discussion

We compared the frequency and nature of early post-transplant complications in ABOi versus ABOc recipients of a living kidney. Our findings are threefold: (*i*) ABOi patients had significantly more postoperative bleeding complications and, therefore, needed significantly more blood transfusions compared to ABOc patients; (*ii*) ABOi patients had significantly more infectious complications, namely significantly more BKV infections, and (*iii*) ABOi patients had a similar number of surgical complications as the ABOc patients.

The originality of our study is that the same medical team was in charge of both the preoperative and postoperative periods. Consequently, we were able to give extensive preoperative attention to prevent and/or correct any coagulation disorders.

In our series, apheresis techniques used during the desensitization period were based on plasmapheresis, DFPP, semi-specific immunoadsorption, specific immunoadsorption, or a combination of any two of these techniques.

It is well known that both plasmapheresis (18) and DFPP (13) lead to fibrinogen depletion, whereas immunoadsorption does not 18 ). Moreover, Hanafusa et al have shown that DFPP and to a lesser extent plasmapheresis lead to factor-XIII depletion, which may have increased the risk of perioperative bleeding. Conversely, when DFPP-treated patients were infused with fresh frozen plasma at the end of the DFPP session that preceded kidney transplantation, this corrected (to some extent) the factor-XIII deficiency (19).

In our series, 20 ABOi patients (40.4%) presented with a perioperative bleed compared to only 8 ABOc patients (18.2%; *P *= 0.006). These incidences in ABOi patients included two cases of intraoperative renal-artery anastomosis leakage and 12 intraoperative bleeds. These incidences required significantly more red blood-cell transfusions in ABOi patients (3.5 ± 3.4) compared to only 0.8 ± 1.9 transfusions in ABOc patients (*P *= 0.0002). Hence, 14 ABOi recipients required an intraoperative blood transfusion compared to only three ABOc patients (*P *= 0.006).

We always try to avoid conducting plasmapheresis on the day before transplantation: if we have to, then we always use fresh frozen plasma as the replacement solution. DFPP is never performed on the day before transplantation. Despite these precautions, at immediate pre-transplant, ABOi patients had significantly lower levels of fibrinogen, platelet counts, prothrombin time, activated cephalin time, and Hb levels compared to ABOc recipients. All these factors contributed to the perioperative bleeds.

The US Renal Data System (USRDS) found that ABOi patients experienced twice the adjusted risk of early hemorrhage (adjusted hazard ratio [aHR] 1.96; 95% CI, 1.19–3.24) compared to ABOc recipients (8).

A Dutch group compared 65 consecutive ABOi patients who had been desensitized using DFPP/immunoadsorption with 130 ABOc live kidney-transplant patients. They found that the ABOi patients needed more red blood-cell transfusions (29% versus 12%, respectively; *P *= 0.005), that intraoperative blood loss was higher in ABOi patients (544 vs. 355 ml, respectively; *P *< 0.005), and that ABOi patients experienced more major bleeds (≥3 red blood-cell transfusions within 24 hours; i.e., 15% versus 2%, respectively; *P *< 0.0005). In multivariate analyses, only the number of preoperative immunoadsorption sessions was associated with the number of red blood-cell transfusions (OR per immunoadsorption session 1.9; *P *< 0.05) (11).

Recently, Kim et al assessed 70 consecutive ABOi patients for the risk of a postoperative bleeding (9). Thirteen percent of these patients had a bleeding episode. Risk factors included a low platelet count <100 000/µl after preoperative plasmapheresis (*P *= 0.02), prolonged activated partial thromboplastin time (*P *= 0.007), impaired platelet function (*P *= 0.02), high pre-transplant blood urea nitrogen (BUN) level (*P *= 0.02), pre-emptive kidney transplantation (*P *= 0.01), and a high anti-ABO antibody titer after plasmapheresis (*P *= 0.02).

From these studies and ours, we conclude that it is of utmost importance to correct potential modifiable factors, such as BUN, by increasing the duration of hemodialysis sessions, improving coagulation factors by transfusing pre-transplant fresh frozen plasma, and by limiting the number of pre-transplant apheresis sessions (if possible), i.e., to target isoagglutinin pre-transplant titers to ≤1/16 (if possible).

Limiting the risk of a perioperative bleed is important because of the subsequent need for a blood transfusion, which, despite immunosuppression, can cause HLA sensitization and antibody-mediated rejection (20).

We found that our ABOi patients experienced significantly more early post-transplant infectious complications compared to ABOc recipients, and this was mainly driven by BKV infection (defined by at least one episode of BKV viremia). Recently, Schachtner et al, reported that ABOi patients had an increased risk of T-cell-dependent infection compared to ABOc patients (21). This might have been because pre-transplant desensitization was associated with the elimination of B-cells, which serve as antigen-presenting cells, thus causing impaired T-cell activation. In their study, ABOi patients were more likely to develop CMV infection, BKV-associated nephropathy and severe sepsis (*P *= 0.001). In addition, ABOi patients had more seriously impaired BKV- and CMV-specific T-cell immunity (*P *< 0.05), as well as decreased CD3+, CD4+ T-cell counts, interferon gamma, and IL-10 levels.

Recently, Sharif et al (15) have shown that, despite similar pre-transplant desensitization protocols and post-transplant immunosuppression therapies, ABOi patients had significantly more BKV-associated nephropathy (BKVAN) than HLA-incompatible patients (17.7% versus 5.9%, respectively; *P *= 0.008). In logistic regression analyses, ABO-incompatibility and age were independent predictive factors for BKVAN. Moreover, protocol biopsies showing C4d deposition without histological features of glomerulitis and capillaritis (a graft accommodation-like phenotype) were significantly less frequent in ABOi patients with BKVAN than in ABOi patients without BKVAN (*P *= 0.04)(15).

In a series of 21 ABOi patients, Habicht et al found that compared to ABOc living-kidney patients, there were significantly more infectious complications, and these were mainly driven by BKV infection (14). In the USRDS study, at early post-transplant (<D90), ABOi patients, compared to ABOc patients with a living kidney, had significantly more wound infections (12.7% versus 7.3%, respectively), more cases of pneumonia (7.6% versus 3.8%, respectively), and more urinary-tract infections and pyelonephritis (24.1% versus 15.3%, respectively). Moreover, in adjusted models, ABO-incompatibility was associated with twice the risk of pneumonia (aHR 2.2, 95% CI: 1.14–4.33) and a 56% higher risk of a urinary-tract infection or pyelonephritis (aHR 1.56; 95% CI: 1.05–2.30) in the first 90 days post-transplant (8). We did not observe these complications, possibly because we used trimethoprim/sulfamethoxazole prophylaxis for the first 6 months post-transplant.

With regards to surgical complications, e.g., renal-vessel thrombosis, urinary leakage, lymphoceles, or wound dehiscence, we did not find any significant difference between ABOi patients and ABOc patients. Conversely in the USRDS study, ABOi patients beyond post-transplant day 90 had 3.15 times more wound infections (aHR 3.55; 95% CI: 1.92–6.57)(8). In addition, Habicht et al, found a significantly greater incidence of lymphoceles in ABOi patients compared to ABOc patients (14).

## 6. Conclusions

In conclusion, our study demonstrates that ABOi kidney-transplant recipients have an outcome that is as good as that of living-kidney ABOc patients; however, within 6 months post-transplantation, ABOi patients present with significantly more infectious complications, such as BKV infection. Also, in the perioperative period, ABOi patients had significantly more bleeding episodes that subsequently required a blood transfusion, although this could be minimized by more careful pre-surgical management of coagulation.

## 7. Limitations of the study

This is a single center study with a limited number of patients. In addition, we did not assess factor XIII pre- and post-apheresis sessions.

## Authors’ contribution

HNB and ZA collected the data; AA performed the apheresis sessions; FS, XG, and ND performed the kidney transplants; LE, AD, NK, and LR recruited and followed-up the patients, and BD performed the isoagglutinin tests. LR wrote the paper. LR edited the final draft. All authors signed the manuscript.

## Conflicts of interest

The authors declared no competing interests.

## Funding/Support

None.

## References

[1] Cecka JM (2001). The UNOS renal transplant registry. Clin Transpl.

[2] Chkhotua A (2012). Paired kidney donation: outcomes, limitations, and future perspectives. Transplant Proc.

[3] De Klerk M, Van Der Deijl WM, Witvliet MD, Haase-Kromwijk BJ, Claas FH (2010). The optimal chain length for kidney paired exchanges: an analysis of the Dutch program. Transpl Int.

[4] Opelz G, Morath C, Süsal C, Tran TH, Zeier M, Döhler B (2015). Three-year outcomes following 1420 ABO-incompatible living-donor kidney transplants performed after ABO antibody reduction: results from 101. Transplantation.

[5] Tanabe K, Ishida H, Inui M, Okumi M, Shirakawa H, Shimizu T (2013). ABO-incompatible kidney transplantation: long-term outcomes. Clin Transpl.

[6] Crew RJ, Ratner LE (2010). ABO-incompatible kidney transplantation: current practice and the decade ahead. Curr Opin Organ Transplant.

[7] Aikawa A, Saito K, Takahashi K (2015). Trends in ABO-incompatible kidney transplantation. Exp Clin Transplant.

[8] Lentine KL, Axelrod D, Klein C, Simpkins C, Xiao H, Schnitzler MA (2014). Early clinical complications after ABO-incompatible live-donor kidney transplantation: a national study of Medicare-insured recipients. Transplantation.

[9] Kim MH, Jun KW, Hwang JK, Kim JI, Chung BH, Choi BS (2015). Risk factors for postoperative bleeding in ABO-incompatible kidney transplantation. Clin Transplant.

[10] Zschiedrich S, Kramer-Zucker A, Jänigen B, Seidl M, Emmerich F, Pisarski P (2015). An update on ABO-incompatible kidney transplantation. Transpl Int.

[11] de Weerd AE, van Agteren M, Leebeek FW, Ijzermans JN, Weimar W, Betjes MG (2015). ABO-incompatible kidney transplant recipients have a higher bleeding risk after antigen-specific immunoadsorption. Transpl Int.

[12] Ohdan H (2015). How can we minimize bleeding complications in ABO-incompatible kidney transplant recipients?. Transpl Int.

[13] Yeh JH, Chiu HC (2001). Coagulation abnormalities in serial double-filtration plasmapheresis. J Clin Apher.

[14] Habicht A, Bröker V, Blume C, Lorenzen J, Schiffer M, Richter N (2011). Increase of infectious complications in ABO-incompatible kidney transplant recipients--a single centre experience. Nephrol Dial Transplant.

[15] Sharif A, Alachkar N, Bagnasco S, Geetha D, Gupta G, Womer K (2012). Incidence and outcomes of BK virus allograft nephropathy among ABO- and HLA-incompatible kidney transplant recipients. Clin J Am Soc Nephrol.

[16] Hwang JK, Kim YK, Kim JM, Chung BH, Choi BS, Yang CW (2013). Comparative analysis of ABO-incompatible living donor kidney transplantation with ABO-compatible grafts: a single-center experience in Korea. Transplant Proc.

[17] Bentall A, Neil D, Sharif A, Ball S (2015). ABO-incompatible kidney transplantation is a novel risk factor for BK nephropathy. Transplantation.

[18] Zöllner S, Pablik E, Druml W, Derfler K, Rees A, Biesenbach P (2014). Fibrinogen reduction and bleeding complications in plasma exchange, immunoadsorption and a combination of the two. Blood Purif.

[19] Hanafusa N, Hamasaki Y, Kawarasaki H, Kido R, Shibagaki Y, Ishikawa A (2013). The effect of different apheresis modalities on coagulation factor XIII level during antibody removal in ABO-blood type incompatible living related renal transplantation. Transfus Apher Sci.

[20] Fidler S, Swaminathan R, Lim W, Ferrari P, Witt C, Christiansen FT (2013). Peri-operative third party red blood cell transfusion in renal transplantation and the risk of antibody-mediated rejection and graft loss. Transpl Immunol.

[21] Schachtner T, Stein M, Reinke P (2015). ABO desensitization affects cellular immunity and infection control after renal transplantation. Transpl Int.

